# Active Factors in the Adult Pig Colon: Microbial Transplantation Versus Supplementation with Metabolites in Weaned Piglets

**DOI:** 10.3390/microorganisms13112533

**Published:** 2025-11-05

**Authors:** Jianhao Cui, Liefa Tang, Zixuan Li, Shuang Wang, Jiayi Zhou, Huichao Yan, Xiaofan Wang

**Affiliations:** State Key Laboratory of Swine and Poultry Breeding Industry, Guangdong Provincial Key Laboratory of Animal Nutrition Control, National Engineering Research Center for Breeding Swine Industry, College of Animal Science, South China Agricultural University, Guangzhou 510642, China; cjh96@stu.scau.edu.cn (J.C.); 17877758271@163.com (L.T.); zixuanli@stu.scau.edu.cn (Z.L.); ws123@stu.scau.edu.cn (S.W.); jyzhou@scau.edu.cn (J.Z.); yanhc@scau.edu.cn (H.Y.)

**Keywords:** cross-age transplantation, tight junction, metabolite, microbiota

## Abstract

The adult pig intestinal microbiota boosts piglet intestinal and microbiome development, thereby improving growth. However, the functional bacteria, metabolites, and their region-specific intestinal roles remain to be characterized. Administration of adult colon microbiota (CM; devoid of metabolites) to piglets promoted intestinal development post-weaning, as indicated by increased intestinal mucosal weight, villus-to-crypt ratio of the ileum (*p* < 0.05), and stimulated mucin secretion (*p* < 0.05). This effect was potentially mediated by modulating beneficial microbiota, including ASV50_*Prevotella* 7, ASV52_*Prevotella* 1, and ASV81_*Coprococcus* 1. Adult colon-derived microbiota was found to preferentially colonize the piglet colon, supported by significantly higher bacterial loads in colonic contents. Piglets receiving adult colon supernatant (CS; without bacterial cells) showed improved feed efficiency (FE; *p* < 0.05), with numerically higher body weight (BW) and average daily gain (ADG) compared to the control (CON) group. Additionally, CS transplantation (CST) promoted intestinal development, potentially by modulating abundances of beneficial bacteria species, including ASV95_*Turicibacter*, and ASV109_*Ruminococcaceae*, which correlated with increased production of antioxidant and anti-inflammatory chemicals, including protocatechuic acid (PCA, *p* < 0.01). Adult colon-derived microbiota and metabolites enhanced intestinal development in piglets. CS supplementation improved growth and immunity, mitigating post-weaning stress potentially through enriching growth-linked bacteria (e.g., *Turicibacter* and *Ruminococcaceae*) and metabolites production (e.g., prephenate and PCA). These findings highlight these functional microbiota and metabolites as promising direct-fed microbial or metabolite additives for piglet growth and intestinal health post-weaning.

## 1. Introduction

The development of the gut microbiota is closely related to the host’s health status and physiological functions [1]. After birth, the gut microbiota undergoes progressive restructuring, stabilization, and functional maturation [2,3,4]. In adult pigs, the phylogenetically mature gut microbiota plays a key role in metabolic optimization, nutrient uptake, pathogen defense, and homeostasis maintenance under perturbation [5,6,7].

Substantial evidence demonstrates that fecal microbiota transplantation (FMT) from adult pigs to weaned piglets has significant positive impacts. These studies show that FMT increases intestinal microbiota diversity, enriches beneficial bacterial genera, and improves intestinal morphology and barrier function, ultimately reducing the incidence of diarrhea [8,9,10]. However, most of these functional microbes and their active derivatives remain largely unidentified.

The intestinal microbial composition varies significantly between different gut segments [11]. The colon serves as a more favorable microbial niche than the small intestine, functioning as the primary site for microbial fermentation and supporting a higher density of bacterial colonization. Notably, colonic microbiota exerts a stronger influence on piglet intestinal health and microbial establishment than the fecal microbiota [10]. Despite its physiological importance, the functional consequences of transplanting colonic microbiota into recipients, particularly its differential effects on the small versus large intestine, remain poorly characterized.

Recent studies have increasingly revealed that bacterial metabolites, including bile acids, short-chain fatty acids (SCFA), and indole derivatives, act as key mediators linking microbial communities to host physiological functions [12,13,14,15,16,17,18,19]. The colon harbors a diverse array of these metabolites that mechanistically affect intestinal health. However, current FMT studies often fail to distinguish between microbial metabolic effects and direct bacterial activities, creating a critical gap in understanding intervention mechanisms. Defining the specific microbial metabolites responsible for the therapeutic effects could revolutionize intestinal health interventions, as it would allow the replacement of complex microbiota transfers with defined biochemical formulations.

Furthermore, rigorous donor selection is essential for FMT, as inadequate screening can pose risks. Failure to eliminate pathogenic bacteria or harmful metabolites from the fecal suspension could compromise intestinal barrier integrity. This compromise is evidenced by the downregulation of tight junction proteins, toll-like receptors [20], and mucin 2 [21,22], and it can trigger inflammatory responses, such as elevating tumor necrosis factor-α and interleukin-6 [10]. Therefore, precise identification of functionally active microbial components is essential to prevent the transfer of detrimental substances.

Based on these considerations, this study was conducted to explore the effects of colonic microorganisms and their metabolites from adult pigs on the intestinal tract and growth phenotypes of piglets and to identify preliminary specific microorganisms or metabolites that will lead to future mechanism exploration. We hypothesized that administrating adult colon microbiota and its metabolite supernatant would exert distinct yet beneficial effects on host piglets, and that each fraction contains specific microbes or metabolites that can be exploited to develop probiotic additives for improving intestinal health.

## 2. Materials and Methods

### 2.1. Animal Ethics Statement

The experiment was performed according to the Guidelines for the Care and Use of Laboratory Animals of South China Agricultural University (2021–0136, Guangzhou, China).

### 2.2. Animal Experimental Design and Sample Collection

**Figure 1:**
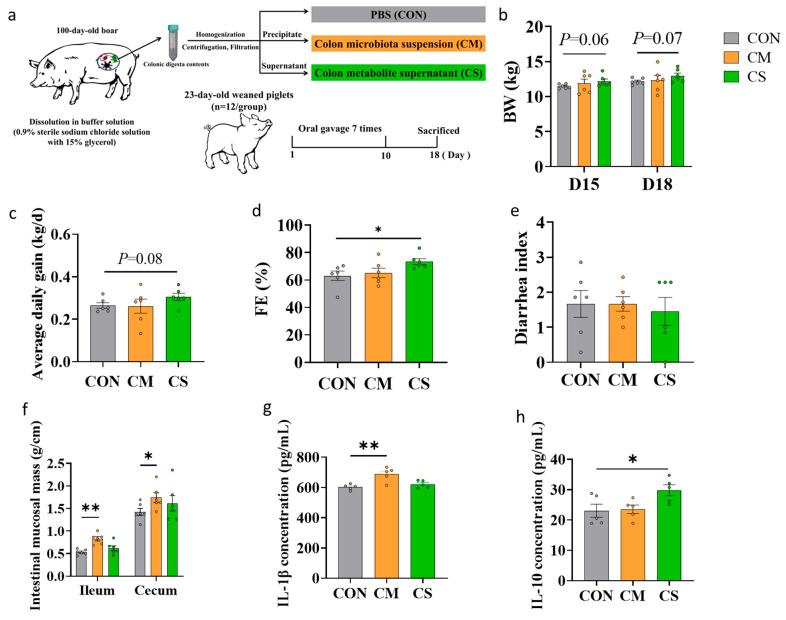
Effects on the growth performance of piglets. (**a**) A total of 36 pigs of weaning age were stratified by initial body weight (BW) and randomly divided into three groups (n = 12/group): (1) CON (control), orally administered PBS; (2) CM, receiving colon microbiota suspension; and (3) CS, given colon metabolite suspension. Each piglet was inoculated orally with 4 mL of suspension on each transplantation day. (**b**–**e**) Effects of colon microbiota transplantation (CMT) and colon supernatant transplantation (CST) on body weight (BW) on the day 15 and 18 (**b**); average daily gain (ADG) (**c**), feed efficiency (FE) (**d**), and diarrhea index (**e**) at the end of trial (n = 6). (**f**) Ileal and cecal mucosal mass were determined after separation from the intestinal segments in piglets (n = 6). (**g**,**h**) Inflammatory factors (IL-1β and IL-10) in the blood of piglets on day 18 (n = 6). * *p* < 0.05. ** *p* < 0.01.

A total of 36 castrated male piglets (Duroc × Landrace × Yorkshire) with a weaning body weight (BW) of 7.75 ± 0.62 kg were purchased from a commercial pig farm in Guangzhou, China. Piglets were randomly divided into three groups (n = 12/group): (1) control (CON), orally administered phosphate-buffered saline (PBS); (2) CM, receiving colon microbiota suspension; and (3) CS, given colon metabolite suspension. Each piglet was administered by gavage 7 times within a 10-day period. Specifically, on each FMT day, each piglet was orally inoculated with 4 mL of suspension (Figure 1a). Diet (Table S1) and housing strategies were uniformly controlled. During the 18-day trial, feed intake was recorded daily, and BW was measured weekly. On the final experimental day, six randomly selected piglets per group were fasted for 12 h following standard protocols before slaughter. Tissues and contents of jejunum, ileum, cecum, colon were systematically collected for downstream analyses. On day 18 of the experimental period, anal swab samples and fresh fecal samples were collected from all piglets and stored at −80 °C.

### 2.3. Preparation of Colon Microbiota and Supernatant Suspension

The gavage solutions were prepared according to a modified protocol [2,11]. A healthy 100-day-old “Duroc × Landrace × Yorkshire” crossbred boar weighing 120 kg was selected as the donor according to the criteria described by Hu et al. [23]. After slaughter, 500 g of colonic contents were collected for the preparation of the colon microbiota and metabolite supernatant. Specifically, colon digesta was suspended in 0.9% sterile NaCl solution containing 15% glycerol at a 1:10 (*w*/*v*) ratio (2 g digesta: 20 mL solution) in a sterile micro-porous filtration homogenization bag (HK-C1930, HuanKai Microbial, Guangzhou, China). The mixture was homogenized using a Stomacher^®^ paddle blender (JX-05, Tuohe Electromechanical Technology Co., Ltd., Shanghai, China) at a frequency of 9 Hz for 4 min to ensure complete homogenization. The resulting suspension (containing both bacterial and metabolite fractions) was collected for secondary filtration using a 5 μm filter to remove large particles. The filtrates were then centrifuged (4000× *g*, 10 min, 4 °C) to obtain bacterial pellets, which were resuspended in sterile PBS-glycerol solution to prepare the CM suspension at a concentration of 1 × 10^8^ CFU/mL (equivalent to 4 × 10^8^ CFU in the 4 mL gavage volume administered to each piglet). The supernatant collected after centrifugation was filtered through a 0.22 μm needle filter (FPE204013, BIOFIL, Guangzhou, China) to remove bacterial cells, and the obtained filtrate was used as the CS supernatant, which represents mostly the colon microbiota-derived metabolites. The prepared gavage fluids (CM and CS) were aliquoted into 50 mL centrifuge tubes and stored at −80 °C for future use.

### 2.4. Serum Inflammatory Factor Evaluation Using ELISA

Blood samples were obtained via anterior vena cava venipuncture, with serum separated by centrifugation (3000× *g*, 15 min, 4 °C) and stored at −80 °C for subsequent biochemical analysis before slaughter. Serum levels of interleukin-1 beta (IL-1β) and interleukin-10 (IL-10) were quantified using commercial ELISA kits (MM-0422O1, MM-0425O1, Jiangsu Enzyme Exemption Industry Co., Ltd., Yancheng, China).

### 2.5. Paraffin Sectioning and Staining

Intestinal samples from piglets were fixed with 4% paraformaldehyde, followed by dehydration and paraffin embedding. The sections were prepared and subsequently stained with hematoxylin and eosin (H&E). Photomicrographs were obtained using an optical microscopy system (XD, SOPTOP, Ningbo, China). Quantitative measurement of ileal villi height and crypt depth were conducted with Image View (version 4.11.18012).

### 2.6. Immunofluorescence Staining

Intestinal samples from piglets were fixed with 4% paraformaldehyde, followed by dehydration and paraffin embedding. The sections were treated with 1% Triton-X100 (#TC281, MIKX, Shenzhen, China) for 10 min and blocked in 10% bovine serum albumin (BSA) for 2 h and then incubated overnight at 4 °C with rabbit anti-TSC2 (1:800; #4308, Cell Signaling Technology, Danvers, MA, USA). Subsequently, the sections were incubated with anti-Olfactomedin 4 (Olfm4) antibody (1:200; #14369Sm Cell Signaling Technology, Danvers, MA, USA), anti-SRY-Box transcription factor 9 (SOX9) antibody (1:200; #380995, ZEN-BIOSCIENCE, Chengdu, China), and anti-MUC2 antibody (1:200; #A14659, ABclonal technology, Wuhan, China) in the dark for 2 h. Cell nuclei were counterstained with DAPI (1:1000) for 10 min. Images were captured using an inverted fluorescence microscope (XD, SOPTOP, Ningbo, China), and the number of positive cells and immunofluorescence intensity were quantitatively analyzed using Image J (version 1.8.0.122) software.

### 2.7. Western Blotting

Total protein was extracted from ileal and colon tissues using radio-immunoprecipitation assay lysis buffer (#DB258, MIKX, Shenzhen, China) and phenylmethanesulfonyl fluoride (#ES-8148, MIKX, Shenzhen, China). Protein concentration was quantified using a BCA Protein Assay Kit (#DB307, MIKX, Shenzhen, China). The samples were diluted with 5 × protein loading buffer, mixed thoroughly, and denatured by boiling in a water bath for 10 min. The denatured proteins and pre-stained markers were separated by sodium dodecyl sulfate-polyacrylamide gel electrophoresis (#P2012, New Cell & Molecular Biotech, Suzhou, China), followed by electrophoretic transfer to a polyvinylidene fluoride membrane. The membrane was sequentially incubated with primary and secondary antibodies, followed by treatment with chemiluminescent reagents (#MK-S500, MIKX, Shenzhen, China). Protein signals were visualized by exposure using a FluorChem M imaging system (ProteinSimple, Inc., Santa Clara, CA, USA).

### 2.8. Isolation of Crypt and Organoid Culture

Five cm jejunal segments were longitudinally incised, and contents rinsed off with pre-cooled DPBS. Intestinal villi were gently scraped with a glass slide, washed with DPBS twice to remove residues. Jejunal segments were cut into 1 cm pieces, incubated with ice-cold soaking buffer, and shaken to detach crypts. Crypts were incubated in DMEM (Cat. No. 11965092, Thermo Fisher Scientific, Shanghai, China) supplemented with 10% FBS (Cat. No. 10099-141, Thermo Fisher Scientific, Shanghai, China) and 1% penicillin-streptomycin, resuspended in ice-cold matrigel, and seeded into pre-coated 48-well plates. After incubating at 37 °C for 10–15 min to solidify the Matrigel, 250 μL of complete intestinal organoid medium was added. The cultures were maintained in a 37 °C, 5% CO_2_ incubator with medium changes every 48 h. Jejunal organoid growth was monitored using an inverted microscope (NIS-Elements, Nikon, Melville, NY, USA). The budding rate was defined as the percentage of organoids within a fixed microscopic field that developed distinct bud-like protrusions between Day 1 and 5.

### 2.9. Absolute Quantification of Colonic Microbes by Flow Cytometry

Colonic contents (2 g) were homogenized in 18 mL of 0.9% sterile saline and centrifuged to obtain a pellet. The pellet was then resuspended in 10 mL of the same saline solution. Subsequently, the resuspended sample was filtered through a 5 μm filter to remove large particles and other impurities, obtaining the bacterial pellet from the colonic contents. The pellet was serially diluted to 10^−4^ dilution, and SYBR Green I staining (1 μL/mL, diluted 1:100 with sterile DMSO) was added. The mixture was incubated at 37 °C in the dark. Then, 500 μL of the incubated sample was analyzed using a flow cytometer (XTG™-1600, Xitogen Technologies, Suzhou, China). The green fluorescence (FL1) and red fluorescence (FL3) signals were collected with bandpass filters centered at 533 nm and >670 nm, respectively. A gating strategy was established and verified using negative controls prepared with 0.22 μm filtered samples. The sample flow rate was set at 30 μL/min, and the thresh The absolute number of bacteria per gram of the original colonic contents was calculated based on the number of particles captured per minute for each sample, the sample flow rate, and the dilution factor before sample processing.

### 2.10. Colonic and Fecal Microbial Analysis by 16S rRNA Amplicon Sequencing

Microbial DNA from intestinal and rectal swabs (n = 6) was subjected to 16S rRNA sequencing. Total microbial DNA was extracted from intestinal contents and feces using the E.Z.N.A.^®^ soil DNA kit (Omega Bio-Tek, Norcross, GA, USA). The quality of the extracted DNA was evaluated by 1% agarose gel electrophoresis, and its concentration and purity were determined using a NanoDrop 2000 (ND-2000, Thermo Fisher Scientific, Shanghai, China). The V3–V4 regions of the bacterial 16S rRNA genes were amplified with the primers 341F (5′-CCTACGGGRSGCAGCAG-3′) and 806R (5′-GGACTACVVGGGTATCTAATC-3′). The fastq reads from the Illumina Nextseq 2000 were processed in QIIME2 platform (version 2022.11) [24]. Paired-end sequences were merged using VSEARCH (version 2.21.1), followed by quality filtering (minimum length ratio set to 1 and minimum quality score set to 30). The Deblur tool was applied with a trimming length of 400 bp and feature table filtering (minimum frequency of 10), while other parameters remained at default values. These features were taxonomically annotated using the SILVA database (version 132). The measurements of alpha, beta diversity, and similarity analysis (ANOSIM) were all performed in QIIME2 (version 2022.11).

### 2.11. Untargeted Metabolomics of Colonic Content Using Liquid Chromatography Mass Spectrometry (LC-MS)

Fifty milligrams of piglet colonic contents (n = 6) were added to an extraction solution (methanol:water = 4:1) containing an internal standard (0.02 mg/mL L-2-chlorophenylalanine). After homogenization (−10 °C, 50 Hz, 6 min), metabolite extraction was carried out by ultrasonication (5 °C, 40 kHz, 30 min). The samples were then incubated at −20 °C for 30 min and centrifuged (13,000× *g*, 15 min, 4 °C). The resulting supernatants were subjected to UHPLC-Q Exactive HF-X metabolomic analysis (Majorbio Bio-pharm Technology Co., Ltd., Shanghai, China). The data were analyzed through the free online platform of majorbio cloud platform (cloud.majorbio.com). Raw data processed with Progenesis QI (version 4.0, Waters Corporation, Milford, CT, USA) were matched against the HMDB, Metlin, and a self-built database. Preprocessed data were subjected to principal component analysis using R’s ropls package (version 1.6.2). Significantly altered metabolites were identified using an Orthogonal Partial Least Squares-Discriminant Analysis (OPLS-DA) model and Student’s *t*-test, with a Variable Importance in Projection (VIP) > 1 and a *p* < 0.05 as selection criteria. Differential metabolites (VIP > 1 and *p* < 0.05) were annotated via KEGG. Pathway enrichment analysis was then performed using Fisher’s exact test (implemented in Python’s (version 3.12.5) scipy.stats package), with significance thresholds of *p* < 0.05 and a false discovery rate (FDR) < 0.1. Pathways were ranked by enrichment factor and −log_10_(*p*).

### 2.12. Data Analysis

Statistical analysis of the results was performed using GraphPad Prism (version 8.0.2) software. All data were presented as mean ± standard error of the mean (SEM). Intergroup comparisons were performed using independent-samples t tests for normally distributed data. Associations were assessed via Pearson correlation analysis. Statistical significance was defined as *p* < 0.05 and a statistical trend was defined as 0.05 ≤ *p* < 0.1.

## 3. Results

### 3.1. Growth Performance of Recipients

Compared to the CON group, CS administration showed a tendency toward increased BW by D18 (+0.83 kg; *p* = 0.07) and increased ADG (*p* = 0.08, Figure 1b,c). In contrast, CS significantly increased FE by 10.3% (*p* < 0.05, Figure 1d). All transplantations did not impact the diarrhea index (Figure 1e). Notably, CM transplantation demonstrated superior effects on intestinal development, significantly increasing mucosal mass in both ileum and cecum compared to the CON group (*p* < 0.05, Figure 1f). Furthermore, the CM group significantly elevated the level of IL-1β (*p* < 0.01, Figure 1g), while the CS group significantly increased IL-10 levels (*p* < 0.05, Figure 1h) compared with the CON group.

### 3.2. Improvement in Small and Large Intestinal Development

**Figure 2:**
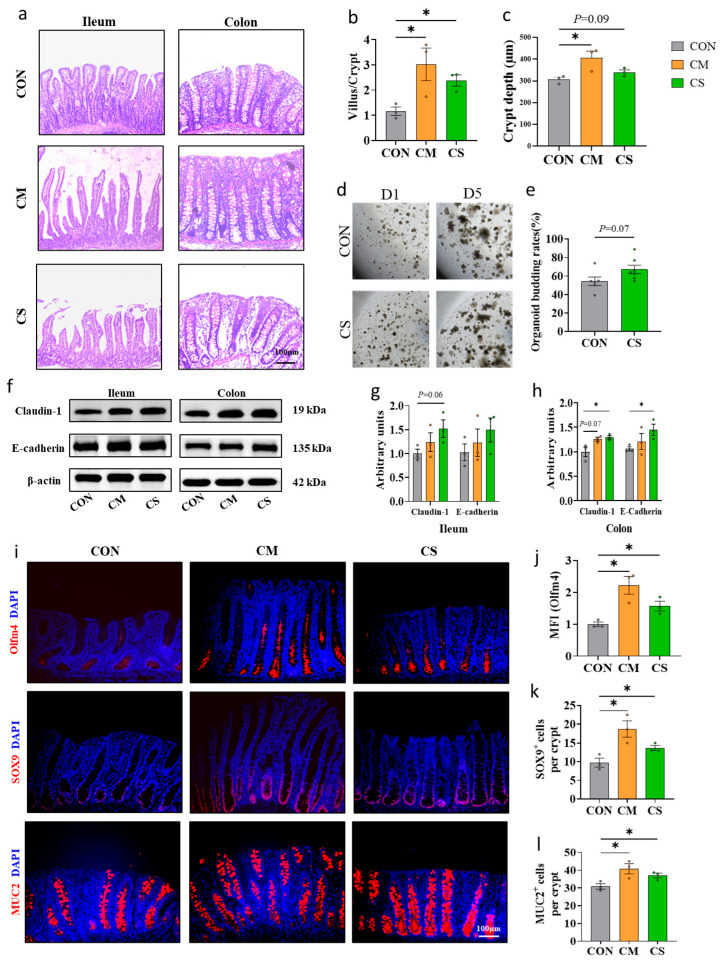
Effects on small and large intestinal development of piglets. (**a**–**c**) Representative images and statistical analysis results of H&E staining of the piglets’ ileum and colon (100× magnification). The villus/crypt of ileum (**b**) and crypt depth of colon (**c**) were measured as indicated in the image (n = 3). (**d**) Representative images of intestinal organoids developed from single crypt cells (40× magnification). (**e**) The organoid budding rates were measured (n = 6). (**f**–**h**) Western blotting analysis of Claudin-1 and E-cadherin protein expression in the ileum and colon of piglets (n = 3). (**i**–**l**) Representative images and statistical analysis results of immunohistochemical staining using antibodies against Olfm4, SOX9 and MUC2 in the colon of piglets (100× magnification), including mean fluorescence intensity (MFI) (n = 3). * *p* < 0.05.

The adult colon microbiota contents, including CM and CS, improved both small and large intestine development in the recipients. Specifically, compared with the CON group, the villus-crypt ratio of the ileum and crypt depth of the colon were significantly increased in CM-fed piglets, while the villus-crypt ratio of the ileum and crypt depth of the colon were also increased in piglets of the CS group (Figure 2a–c). Even without significance, pigs administered CS showed a tendency for 12.8% higher jejunal organoid budding rates compared to the CON group (*p* = 0.07, Figure 2d,e). Furthermore, the abundances of Olfm4 (a marker for intestinal stem cells), SOX9 (differentiated cells), and MUC2 (goblet cells) were all significantly elevated in both the CM and CS groups relative to the CON group (*p* < 0.05, Figure 2i–l). Moreover, both CM and CS administration consistently increased Claudin-1 and E-Cadherin protein expression in piglet ileum and colon (Figure 2f–h). These results demonstrate the beneficial effects of colon microbiota transplantation (CMT) and colon supernatant transplantation (CST) on intestinal development in piglets.

### 3.3. Gut Microbiota Modulation in Recipients

**Figure 3:**
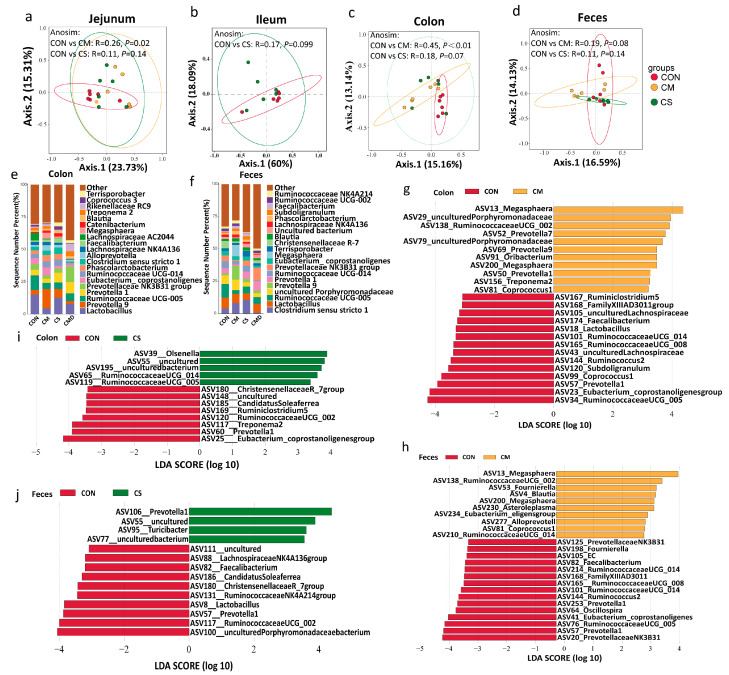
Effects on the gut microbiota of piglets. (**a**–**d**) Principal component analysis (PCoA) scatter plot demonstrating beta diversity among the jejunal, ileal, colonic, and fecal microbiota of CON, CM and CS groups. (**e**,**f**) Stacked bar graph illustrating the actual abundance of colon and feces microbiota at the genus level in CON, CM, CS and colon microbiota donor (CMD) groups. (**g**–**j**) Linear discriminant analysis (LDA) effect size (LEfSe) chart displaying differentially abundant taxa between CON and CM or CS groups in colon and feces.

Compared to the CON group, piglets administered adult colonic microbiota exhibited significantly distinct intestine microbial communities in both the colon (*p* < 0.01) and jejunum (*p* < 0.05), with a trend toward separation in fecal microbiota (*p* = 0.08). Pigs fed CS tended to have distinct colonic microbial communities compared to the CON group (*p* = 0.07; Figure 3a–d). Moreover, the colonic microbiota donor (CMD) was predominantly composed of *Lactobacillus*, *Prevotella*_1, *Ruminococcaceae_UCG-005*, *Prevotellaceae NK3B31 group*, *Rikenellaceae Rc9*, and *Treponema* 2 (Figure 3e). These taxa were also observed in the colonic contents of the CM-treated piglets. In terms of genus abundance, the CM group outperformed the CON group in terms of colonic *Prevotella* 9, *Megasphaera*, *Phascolarctobacterium*, and *Ruminococcaceae UCG_014* abundance, while the dominant group changed from *Lactobacillus*, *Ruminococcaceae UCG_005*, and *Lachnospiraceae AC2044* to *Prevotella* 9 (Figure 3e and Figure S1a). Interestingly, the CM group’s feces showed an increase in *Clostridium sensu stricto* 1 abundance and a significant decrease in *Ruminococcaceae UCG-005* abundance. In contrast, the dominant genera, *Prevotella* 9 and *Megasphaera*, were significantly elevated (Figure 3f and Figure S1c). In addition, *Prevotella* 1, *Ruminococcaceae UCG_014* and *Faecalibacterium* were all increased in the colon of piglets after CST, while colonic *Ruminococcaceae UCG_005* (*p* < 0.05) abundance decreased (Figure 3e and Figure S1b). In addition, CS treatment increased the abundance of *Clostridium sensu stricto* 1 and *Prevotella* 1 in the feces, and the dominant bacteria shifted from *Lactobacillus* and *Ruminococcaceae UCG_005*, which had decreased in abundance to *Clostridium sensu stricto* 1 (Figure 3f and Figure S1d).

The LEfSe analysis revealed that the CM group had significantly higher levels of ASV13_*Megasphaera*, ASV52_*Prevotella* 7, and ASV69_*Prevotella* 9 in the colon and ASV13_*Megasphaera* and ASV81_*Coprococcus* 1 in the feces when compared to the CON group (Figure 3g,h). Furthermore, compared to the CON group, the CS group had considerably higher levels of ASV39_*Olsenella*, ASV65_*Ruminococcaceae UCG_014*, and ASV119_*Ruminococcaceae UCG_005* in the colon, while the feces had significantly higher levels of ASV106_*Prevotella* 1 and ASV95_*Turicibacter* (Figure 3i,j).

### 3.4. Microbiota Variation Enhances Beneficial Metabolism

**Figure 4:**
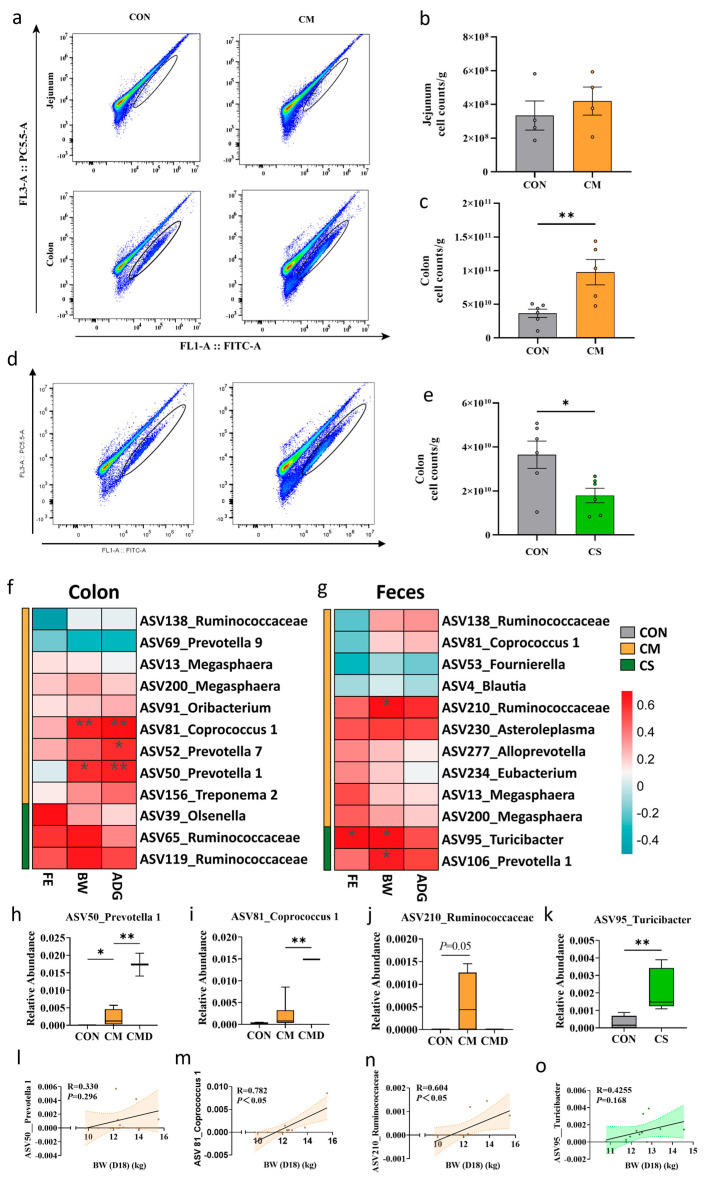
Colonization of transplanted microbiota and its correlation with growth performance. (**a**) Characterization of the number of bacteria in the jejunal and colonic contents using an analytical flow-cytometric panel in CON and CM groups. (**b**,**c**) Statistical analysis results of the number of bacteria in the jejunal (n = 4) and colonic contents (Con: n = 6, CM: n = 5). (**d**) Characterization of the number of bacteria in the colonic contents using an analytical flow-cytometric panel in CON and CS groups. (**e**) Statistical analysis results of the number of bacteria in the colonic contents (n = 6). (**f**,**g**) Heatmap of the correlation between the differential bacteria and piglet growth performance (e.g., FE, BW, ADG) in colon (**f**) and feces (**g**) in CM (yellow) and CS (green) groups. (**h**–**k**) Boxplots depicting the difference in the abundance of the ASV50_*Prevotella* 1, ASV81_*Coprococcus* 1 in colon and ASV210_*Ruminococcaceae* and ASV95_*Turicibacter* in feces. (**l**–**o**) Scatter plots with regression line showing correlations of bacterial ASVs with BW on D18 in the colon (**l**,**m**) and feces (**n**,**o**). * *p* < 0.05. ** *p* < 0.01.

To assess the colonizing efficacy of transplanted adult microbiota, we performed absolute quantification of bacterial cells in weaned piglets’ intestinal contents using flow cytometry. Flow cytometric analysis revealed that the CM group had a significantly higher bacterial load in colonic contents than the CON group (*p* < 0.01), whereas no significant difference was observed in the jejunum (Figure 4a–c). This indicates that the transplanted adult microbiota preferentially colonized the colon over the small intestine. In contrast, the bacterial load in the colonic contents of the CS group was significantly lower than that of the CON group (*p* < 0.05, Figure 4d,e).

We subsequently identified the beneficial microbial drivers in adult donor colon contents that promote growth performance and gut maturation in the young recipients. Colonic ASV50_*Prevotella* 1 and ASV81_*Coprococcus* 1 demonstrated a significant positive correlation with both BW and ADG (Figure 4f,l,m). Additionally, ASV52_*Prevotella* 7, which was significantly enriched in the colon of the CM group, showed a significant positive correlation with ADG and a positive correlation with both BW and FE (Figure 4f and Figure S1e). ASV210_*Ruminococcaceae*, which was significantly enriched in the feces of CM piglets, exhibited strong correlations with BW, ADG, and FE (Figure 4g,n). Furthermore, in the colon of CS piglets, ASV39_*Olsenella*, ASV65_*Ruminococcaceae* and ASV119_*Ruminococcaceae* all exhibited a positive correlation with growth performance (BW and ADG) and FE (Figure 4f). In the feces of CS group, ASV95_*Turicibacter* was significantly correlated with BW and FE (Figure 4g,o). Additionally, ASV106_*Prevotella* 1 represents another potential probiotic improving BW, ADG and FE (Figure 4g). Notably, ASV50, 81, 95 and 106 were potentially colonizers from the donor that strongly correlated to piglets’ growth (Figure 4f–o and Figure S1e–j).

### 3.5. Potential Beneficial Metabolites

**Figure 5:**
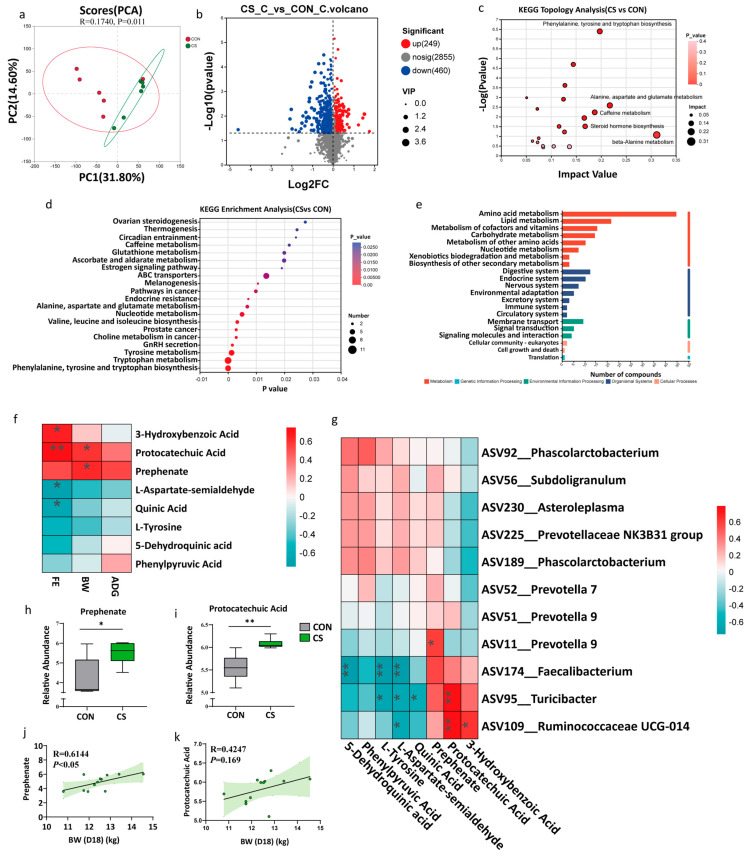
Screening of potentially beneficial metabolites. (**a**) Principal component analysis based on positive and negative colon metabolite profiles. (**b**) Volcano plot of differentially expressed metabolites following colon supernatant treatments (|Fold Change| = 1). (**c**–**e**) KEGG topology analysis, enrichment analysis and pathway classification of the differentially expressed metabolites in the different piglets. (**f**,**g**) Heatmap of the correlation between the differential metabolites and growth phenotypes (**f**) or bacterial (**g**) in the CON and CS groups. (**h**,**i**) Boxplots depicting the difference in the abundance of the prephenate (**h**) and protocatechuic acid (**i**). (**j**,**k**) Scatter plots with regression line showing correlations of prephenate and protocatechuic acid with BW on D18. * *p* < 0.05. ** *p* < 0.01.

Piglets receiving CS supplementation showed improved growth performance, altered gut microbiota structure, and significantly shifted colonic metabolic profiles (*p* = 0.01; Figure 5a). LC-MS analysis identified metabolites present in the CS inoculum itself, such as stercobilin, L-glutamine, oxindole, and others. We further identified 249 metabolites (VIP > 1, *p* < 0.05) that were significantly increased in the CS-administered piglets (Figure 5b) using LC-MS. Specifically, gentisic acid [25] and phenylbutyrylglutamine [26] were enriched in the recipients’ colon (Table S2). Pathway analysis showed strong enrichment in amino acid metabolism, especially phenylalanine, tyrosine and tryptophan biosynthesis, indicating the potential microbial modulation of amino acid metabolism (Figure 5c–e). Among the elevated metabolites, the levels of 3-hydroxybenzoic acid, protocatechuic acid, and prephenate were positively correlated with BW, ADG, and FE (Figure 5f), suggesting their potential importance in promoting growth. Notably, protocatechuic acid, a potentially anti-inflammatory and antioxidant chemical, and prephenate, a precursor substance for the biosynthesis of L-tyrosine and L-phenylalanine [27], were significantly increased in the CS piglets and strongly correlated with the growth of piglets (Figure 5h–k). Thus, we speculated that CS enriched certain bacteria involved in producing these functional chemicals, resulting in the promoted host metabolism and health. Further correlation interpretation suggested ASV95_*Turicibacter*, ASV109_*Ruminococcaceae* UCG-014 and ASV174_*Faecalibacterium* were potentially involved in producing these functional compounds (Figure 5g).

## 4. Discussion

### 4.1. Respective Effects of CMT and CST on Growth Performance and Intestinal Development of Recipient Piglets

While previous FMT studies have focused exclusively on bacterial cell-containing inoculum, the functional contribution of cell-free supernatant components remains unexplored in piglets. Our study primarily revealed that gavage with colon content supernatant alone, devoid of bacterial cells, exerts significant biological effects, highlighting the underestimated role of soluble microbial factors in intestine development. Compared to CON, piglets receiving CST demonstrated significantly improved growth performance and intestinal development. This is consistent with numerous studies that microbial metabolites (e.g., SCFAs and tryptophan metabolites) play a crucial role in maintaining intestinal barrier function and promoting intestinal development and growth performance [15,28,29,30,31,32]. Administering colonic fluid with only bacterial cells (no metabolites) showed significant gut development effects, consistent with conventional FMT outcomes. CM-treated piglets exhibited significant improvements in intestinal structure, an effect potentially mediated by the colonization of adult-derived gut microbiota. This provides novel evidence that early exposure to maturity-associated bacteria promotes intestinal development in young piglets. This contrasts with germ-free models, where the absence of a microbiota leads to impaired gut maturation, immune deficits, and increased disease susceptibility [31,33,34,35], collectively underscoring the indispensable role of microbial communities in maintaining host homeostasis [35,36,37,38,39].

### 4.2. Effects of CMT and CST on the Gut Microbiome of Recipient Piglets

CMT induced the most pronounced shifts in gut microbiota composition, particularly in the colon. Absolute quantitative data revealed that adult colon-derived microbiota colonize the colon in the largest quantity, rather than the jejunum, establishing the colon as the primary colonization site for CM, aligning with prior reports on microbial niche specialization [11]. *Prevotella*, *Coprococcus*, and *Megasphaera* abundances increased significantly after gavage in CM-treated piglets and were identified as potential intestinal colonizers. Importantly, they were shown to be related to piglet BW, ADG, and FE. *Prevotella*, a dominant genus in adult swine microbiota, plays a pivotal role in carbohydrate metabolism and is critical for weaning adaptation. Its extensive repertoire of fiber-degrading enzymes (e.g., xylanases, pectinases) facilitates efficient utilization of plant-based diets, making it a key taxonomic marker for successful transition to solid feed [40,41,42]. Although *Megasphaera* remains understudied in swine gut microbiota, our study suggested it plays critical roles in intestinal health. *Megasphaera*, a major inhabitant of the adult pig intestine [43,44], converts lactate to SCFAs like acetate and propionate [45,46,47]. These SCFAs promote growth [37,48,49,50]. This lactate-utilizing capability may enhance community stability and support epithelial function, positioning *Megasphaera* as a potential keystone taxon in piglet intestinal development [43,47]. Furthermore, *Coprococcus*, a primary SCFA producer, has emerged as a potential key taxon for intestinal health. This bacterium can reshape the microbial community and suppress genes linked to lipid accumulation (e.g., *Fasn*, *Scd1*), inflammation, and fibrosis (e.g., *Tnf-α*, *Ifn-γ*), underscoring its therapeutic potential [17,51]. These findings suggest that post-weaning colonization of these adult-type gut microbiota is pivotal in shaping intestinal development. 

Additionally, *Turicibacter* abundance in the CS group demonstrated significant positive correlations with growth performance parameters, consistent with the probiotic effects reported by Wang et al. (2019) [43]. Notably, emerging evidence suggests *Turicibacter* may exert dual benefits through metabolic modulation (e.g., lipid absorption enhancement) and immunoregulatory functions, as evidenced by its altered colonization patterns in immunodeficient versus wild-type mice [52,53]. This positions *Turicibacter* as a unique microbiota member capable of simultaneously improving growth efficiency and immune homeostasis in piglets. *Ruminococcaceae* plays a key role in intestinal growth and development. Research has demonstrated that *Ruminococcaceae*-produced short-chain fatty acids can mediate α-linolenic acid to stimulate the proliferation of ISCs by triggering the Wnt/β-catenin signaling pathway [54].

### 4.3. Effects of CST on the Colonic Metabolic Profiles of Recipient Piglets

Dietary plant components undergo microbial biotransformation in the intestine, yielding bioactive secondary metabolites that modulate host physiology. Our metabolomic analysis revealed significant enrichment of microbially derived compounds (e.g., PCA, prephenate) in the CS group. They were also correlated with improved growth performance, suggesting that these metabolites may exert a beneficial effect through enhanced nutrient utilization and metabolic regulation. PCA has been widely recognized for its bioactive effects: antibacterial, anti-inflammatory, and antioxidant properties. A study found that dietary supplementation with PCA can lower inflammation and liver damage in a high-fat diet-induced model of metabolically associated fatty liver disease in mice [31]. Furthermore, a dietary supplement with PCA can improve growth performance and immunity of weaned piglets [55]. Consequently, PCA might improve the immunological response between pigs and the microbiota, which would improve piglets’ growth performance. Prephenate is a key intermediate in the biosynthesis of aromatic amino acids, including phenylalanine and tyrosine [27]. Although its biochemical role is well defined, its specific contributions to post-weaning physiology, particularly in intestinal development and nutrient utilization, require further investigation. Critically, we note that the study’s scale and duration limit generalizability, and the proposed microbe-metabolite interactions remain correlative. Consequently, future work is needed to confirm these interactions and define their causal mechanisms.

## 5. Conclusions

Compared with traditional FMT, our study clarified the different effects induced by the transplantation of microorganisms and metabolites. CMT treatment enriched beneficial gut bacteria (*Megasphaera*, *Coprococcus*, and *Prevotella*), which specifically colonized the piglet colon and improved intestinal morphology. In contrast, CST enhanced growth performance and intestinal development primarily through microbial metabolites (PCA and prephenate), demonstrating distinct but complementary mechanisms. These findings highlight the niche-specific microbial and metabolites and their roles in cross-age intestinal health and systemic development.

## Data Availability

The original data presented in the study are openly available in the Sequence Read Archive (SRA) repository, https://www.ncbi.nlm.nih.gov/bioproject/531671 (SRA accession: PRJNA1297990 and PRJNA1303396, available on 1 August 2026).

## Supplementary Materials

The following supporting information can be downloaded at https://www.mdpi.com/article/10.3390/microorganisms13112533/s1, Figure S1: Relative abundance of bacteria; Table S1: Piglet feed formula; Table S2: Top 20 differential metabolites in the colonic metabolite; Table S3: The information of cell markers.
