# Supplementary material for: Active Factors in the Adult Pig Colon: Microbial Transplantation Versus Supplementation with Metabolites in Weaned Piglets
Source: Microorganisms. 2025 Nov 5;13(11):2533. doi: 10.3390/microorganisms13112533 (PMC12654662; doi:10.3390/microorganisms13112533)

Supplemental Table S1 Piglet feed formula

Table S1. Ingredient and chemical composition of the basal diet as fed basis

Ingredient	Content (%)	Nutritional Level	Content (%)
Corn	43.20	Digestible Energy, MJ/kg	14.00
Flour	10.00	Crude Protein	18.36
Fish Meal	2.50	Lysine	1.50
Expanded Soybean	10.00	Methionine + Cystine	0.70
Expanded Soybean Meal	10.00	Threonine	0.85
Soybean Concentrate Protein	5.00	Tryptophan	0.28
Glucose	2.00	Sodium Chloride	0.30
Whey Powder (High Lactose)	10.00	Calcium	0.69
Sucrose	2.50	Phosphorus	0.52
Lysine Hydrochloride	0.60		
Threonine	0.40		
Methionine	0.10		
Tryptophan	0.10		
Calcium Lactate	1.50		
Calcium Hydrogen Phosphate	0.80		
Sodium Chloride	0.30		
1% Premix	1.00		
Total	100.00		

Note: The premix provides per kilogram of feed: Fe 101.00 mg, Mn 42.00 mg, Cu 15.00 mg, Zn 102.00 mg, I 0.60 mg, Se 0.3 mg, VA 10500.00 IU, VD3 2100.00 IU, VK3 3.50 mg, VB1 2.24 mg, VB2 9.80 mg, VB6 2.80 mg, niacin 35 mg, folic acid 1.04 mg, VB12 0.05 mg, and VE 30 mg. The crude protein content is a measured value, while other values are calculated ones.

Supplemental Table S2 Top 20 differential metabolites in the colonic metabolite

Table S2 Top 20 differential metabolite in the colonic metabolite

Metabolite
(7Z)-Tetradec-7-enedioylcarnitine
Physoperuvine
Phenylbutyrylglutamine
Hernandulcin
Tropine
3,3'-Dihydroxy-4',5,7-trimethoxyflavan
Terpestacin
(3R)-3,4-Dihydroxy-3-(hydroxymethyl) butanenitrile 4-glucoside
1-Hydroxy-2,2,5,5-tetramethyl-2,5-dihydro-1h-pyrrole-3-carboxamide
Isopropyl beta-D-glucoside
Deoxycholylalanine
4-[(2,4-Dihydroxy-3,3-dimethylbutanoyl) amino] butanoic acid
N-Methyl-2-pyrrolidinone
Baccatin III
Lincomycin
Gentisic Acid
DG (20:4(8Z,11Z,14Z,17Z)/16:0/0:0)
Beta-Alanine
Norhygrine
Tert-Butyl (2-(2-hydroxyethoxy)ethyl)carbamate

Supplemental Table S3 The information of cell markers

Table S3 The information of cell markers

Name of the cell markers	Represent cells	Function
Olfm4	Intestinal Stem Cells	Maintains the self-renewal ability of intestinal stem cells and preserves their undifferentiated stem cell properties
SOX9	Stem/progenitor cells and Paneth cells	Maintains the intestinal stem cell pool and directs their lineage differentiation.
MUC2	Goblet cells	Major mucus structural protein; provides a critical barrier between commensals/pathogens and the epithelium.

Figure S1. Relative abundance of bacteria

(a-d) Boxplots depicting the difference in the abundance of the differential bacteria in colon and feces of each group.

(e-j) Boxplots depicting the difference in the abundance of the bacterial ASVs in colon. (e-h) and feces (i-j) of each group. **P* < 0.05. ***P* < 0.01

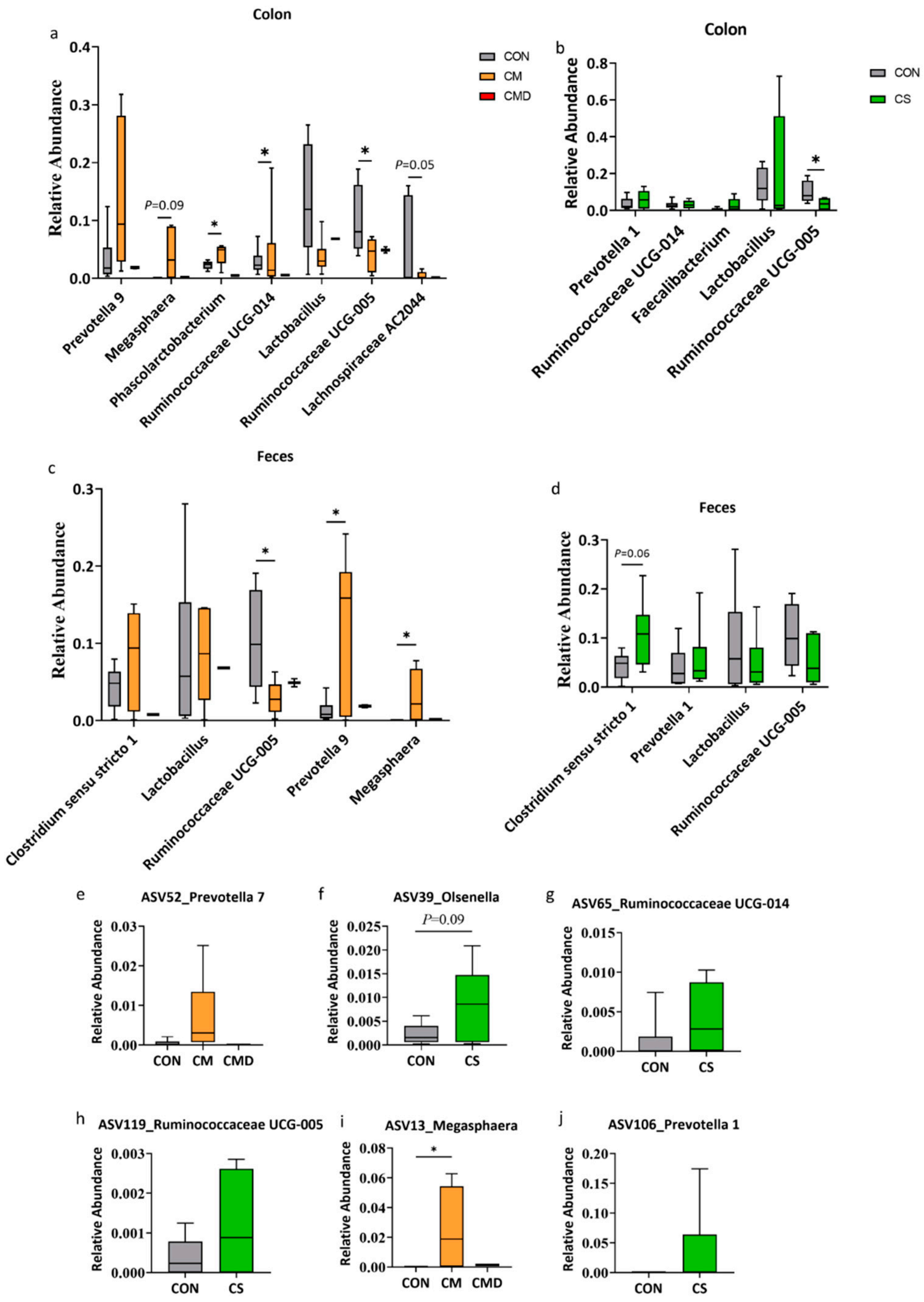

Supplement: Supplementary file 1 [file microorganisms-13-02533-s001.zip › microorganisms-3871110-supplementary.pdf]
